# The SomnuSeal Oral Mask Is Reasonably Tolerated by Otherwise CPAP Noncompliant Patients with OSA

**DOI:** 10.1155/2013/840723

**Published:** 2013-10-08

**Authors:** N. Katz, Y. Adir, T. Etzioni, E. Kurtz, G. Pillar

**Affiliations:** ^1^Sleep Laboratory, Assuta Medical Services and Wolfson Hospital, 69710 Holon, Israel; ^2^Pulmonary Unit, Carmel Hospital and Clalit Health Care, 34362 Haifa, Israel; ^3^Sleep Clinic, Clalit Health Care, 34362 Haifa, Israel; ^4^Sleep Laboratory, Rambam Health Care Campus, P.O. Box 9602, 31096 Haifa, Israel; ^5^Department of Pediatrics, Carmel Hospital and Faculty of Medicine, Technion-Israel Institute of Technology, 34362 Haifa, Israel

## Abstract

Compliance with CPAP is the major limiting factor in treating patients with OSA. The novel SomnuSeal mask is an oral self-adaptable mask located between the teeth and the lips ensuring that there are no air leaks or skin abrasions. Fifty patients with AHI > 20, who failed previous CPAP trials, were asked to sleep with the mask for one month. In all patients, the mask was connected to an AutoPAP machine with a heated humidifier. Efficacy, convenience, and compliance (average usage for 4 or more hours per night) were monitored. Fifty patients (41 m and 9 f, mean age 57 ± 12 years, BMI 33.6 ± 4.9 kg/m^2^, and AHI 47 ± 23/h) participated. Eleven were classified as compliant (average mask usage of 26 nights, 4.7 hours per night), five were only partially compliant (average usage of 13 nights, 2.9 hours per night), and 34 could not comply with it. In all patients who slept with it, the efficacy (assessed by residual AHI derived from the CPAP device) was good with an AHI of less than 8/hour. Interestingly, the required optimal pressure decreased from an average of 9.3 cmH_2_O to 4.6 cmH_2_O. The SomnuSeal oral interface is effective and may result in converting noncompliant untreated patients with OSA into well-treated ones.

## 1. Introduction 

Obstructive sleep apnea (OSA) is a common disorder characterized by recurrent hypoxemia, hypercapnia, and arousal from sleep and is associated with adverse neurocognitive and cardiovascular sequelae [1–6]. Application of continuous positive airway pressure (CPAP) leads to improvements in many of these adverse parameters [7–9], although residual sleep disordered breathing may still persist [10, 11].

The major limiting factor of CPAP treatment is compliance [12–14].

Some of the most important factors that have been reported as limiting compliance are skin abrasions or eruptions due to the pressure exerted by the mask, mask pressure on the ridge of the nose, claustrophobia, aerophagia, air leaks (eye irritation), dry mouth, dry nose, nasal stuffiness, epistaxis, sinusitis, facial pain or a noisy device, or pressure intolerance [15–21]. Other factors that have been identified as affecting compliance consist of disease severity, daytime sleepiness, motivation, age, socioeconomic status, education, race, marital status, spouse support, and copayment [12–31]. Even with the advanced and newer devices (such as the “C-Flex” CPAP device, BPAP, or automatic CPAP), data are not convincing for improved compliance [32–35].

**Figure 1:**
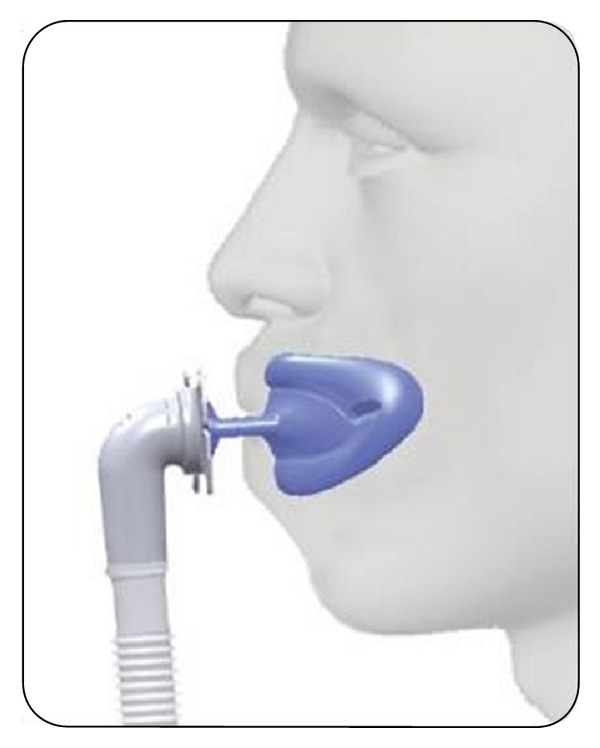
SomnuSeal mask.

Since CPAP treatment has a dramatic beneficial impact on patients [7–9, 23, 36–39], it is of great importance to seek interfaces that can improve compliance. The novel SomnuSeal mask (Figure 1) is an oral self-adaptable mask located between the teeth and the lips, ensuring that there are no air leaks or skin abrasions. It is more comfortable, adjusts better to the patient's specific anatomical structure, and potentially reduces rejection by claustrophobic patients. In a series of preliminary studies (published as abstracts [40, 41]), it has been shown to potentially improve compliance in struggling or otherwise CPAP noncompliant patients. These preliminary studies were conducted on a relatively small number of patients and for relatively short periods of time (one night up to one week of use). However, the results were encouraging, indicating that up to 40% of patients with moderate-severe OSA may comply and tolerate the SomnuSeal mask. We speculated that the oral mask will be a second and not a first line of treatment, and therefore this study was planned to examine a longer period of efficacy and compliance with the SomnuSeal interface (one month of treatment), only in CPAP noncompliant patients with moderate-severe OSA.

## 2. Methods 

### 2.1. Participants

Fifty patients were recruited with moderate-severe OSA (AHI > 20) who were otherwise untreated. All patients were established as noncompliant, having tried at least one CPAP mask previously and failed to comply with it or with any previous CPAP mask they had tried. Inclusion criteria consisted of a previous diagnosis of OSA with an AHI > 20/h, age above 18 years (males or females), failure of at least one CPAP mask in the past, not considering any other treatment (i.e., patients who are otherwise untreated), and consent to participate. All patients were recruited from the patients' registry (archives) of our sleep clinic and gave written informed consent prior to participation. The study was approved by the Rambam Health Care Campus Institutional Review Board (RMB-0010-11). 

Exclusion criteria consisted of any unstable medical condition, active malignancy, age below 18 years, pregnant or lactating women, treatment for OSA other than CPAP (i.e., patients who use dental appliance or consider surgery), and periodontal diseases or mouth lesions according to the investigator's judgment. 

### 2.2. Baseline Polysomnographic Study

All participants had a baseline full night sleep study using electroencephalogram, electrooculogram, submental electromyogram, bilateral anterior tibialis electromyography, electrocardiogram (ECG), nasal-oral airflow (thermistors and nasal pressure), chest and abdominal wall motion (piezo- or impedance belts), body position, and arterial oxygen saturation (Embla, Broomfield, USA). Sleep staging and respiratory indices were scored by a trained technician. Apnea was defined as a ≥90% decrease in airflow persisting for at least 10 sec, while hypopnea was defined as a ≥50% decrease in the airflow amplitude (relative to baseline, persisting for at least 10 sec) with an associated ≥3% oxygen desaturation or an arousal. The apnea hypopnea index (AHI) was calculated as the number of respiratory events (apnea and hypopnea) divided by the total sleep time. 

### 2.3. CPAP Devices and Interface

All participants were fitted in the clinic with CPAP and a SomnuSeal oral mask and were instructed to sleep with it on a nightly basis at home. In the clinic visit a trained sleep technician/respiratory therapist fitted them with the equipment. They were given two sizes of oral masks and were allowed to decide which one was more comfortable. 

The SomnuSeal is an intraoral CPAP interface that provides the needed seal of the oral cavity from outside air (atmosphere) without the shortcomings of other intra- or extraoral masks (Figure 1). The mask is composed of a central part that delivers the compressed air directly to the oral cavity without impingement on intraoral tissues. This central part is surrounded by a special soft silicone part that can engage gently the delicate intraoral tissues in such a way as as to create a peripheral seal so that the intra-oral cavity is secluded from outside atmosphere. The interface is held in place by engagement of the central part with the lingual side of the lips. A nasal peg was not needed. After fitting the mask to a patient, it was connected to an AutoPAP machine (Winemann WM 27460/S). This is a CPAP with a heated humidifier and a usage meter which was reviewed after one month of participation (see below). In addition to hours of use, the device recorded the provided pressure, residual sleep disordered breathing events, and air leaks. 

### 2.4. Study Design

The study consisted of two clinic visits and three telephone visits. In the first clinic visit, the selected patients reported to the clinic and signed an informed consent. A brief physical examination focusing on their oral cavity was then performed by a sleep physician. They were then fitted with the SomnuSeal mask, and, for about 30 minutes, they exercised breathing with it whilst being awake. They were trained for correct mask usage, how to connect it to the AutoPAP machine themselves, how to handle the mask and device in home, and how to wash the mask. They then took the equipment home and were instructed to use it every night for a one-month period. 

During the month of the study, telephone visits were performed on a weekly basis. After one, two, and three weeks of use, a research assistant called each participant for an update. In the call, the participants reported their level of satisfaction and subjective tolerance of the mask. In cases when they needed some extra assistance with the interface or CPAP, the study technician discussed it with them, and upon their request they were also offered an actual meeting with him. 

After one month of use, the second clinic visit (end of study visit) took place, in which another brief physical examination of the subjects was conducted (again focusing on the oral cavity). At that time the subjects were asked to complete a questionnaire evaluating their use of the mask, and the CPAP meter and output were downloaded and examined by the researcher. The outcome measures included the data retrieved from the CPAP objective usage meter so the actual time it was used by the patient was objectively quantified. In addition, efficacy was quantified based on the CPAP internal recorder (i.e., optimal pressure, residual AHI, and leaks). A subjective satisfactory questionnaire was completed by each participant at that visit. Data were collected regarding the convenience of using the mask, difficult/ease of using and handling it, changes in these parameters with time (from the first to last week of usage), potential side effects, self-assessment of usage, and free text of subjective judgment of the mask.

Data from the patients' charts, demographic data, usage meter data driven from the CPAP machine, and data from the satisfaction questionnaires were all collected in a data management sheet (Excel) for statistical analyses.

## 3. Results 

Fifty patients participated in the study. Their mean age was 57 ± 12 years. Their average AHI was 47 ± 23/hour, and their average BMI (body mass index) was 33.6 ± 4.9 kg/m^2^. Forty-one patients were males and 9 were females. 

The patients were categorized into three subgroups according to their compliance with CPAP: compliant patients (usage of the CPAP for more than 70% of the nights, for four hours per night or more), struggling or partially compliant patients (who used it intermittently but in less than required to be considered as a compliant patient), and noncompliant patients (who could not tolerate the device). These results are summarized in Table 1.

The major results obtained from the satisfaction questionnaire are summarized in Table 2 (same classification as in Table 1). As can be seen (and expected), patients who complied with the SomnuSeal mask expressed it in their satisfaction, complained less of excess salivation, and indicated a high index of willingness to obtain the mask. 

The two most importantly reported side effects were excess salivation and inconvenience of the mask in the mouth due to pressure on the lips. However, it should be stated that, in the physical examination at the end of the study, none had any ulcers or bruises on their lips or gingival. 

Of note, the automated supplied air pressure provided by the autoPAP dramatically decreased with the SomnuSeal compared to the nasal mask. While with the nasal mask the average required pressure was 9.3 ± 1.8 cmH_2_O, the pressure required with the SomnuSeal mask was 4.6 ± 0.9 cmH_2_O. 

## 4. Discussion 

The major finding of our study is that 22% of patients with moderate-severe OSA, who failed any other treatment and were noncompliant to at least one CPAP mask which they tried and who otherwise remained untreated, managed to comply with the SomnuSeal oral mask for at least one month of use. They used it on average for 26 nights, with an average of 4.7 hours per night. This can potentially convert them from untreated to reasonably treated patients.

The advantages of CPAP treatment in patients with OSA are well documented. CPAP has been shown to reduce insulin resistance, improve blood pressure, reduce stroke, improve endothelial function, reduce health care utilization, reduce road accidents, improve cognition, improve mood, and improve quality of life [7–9, 23, 36–39]. Despite all of these factors, about 40% of patients who need CPAP remain untreated. The leading cause for CPAP failure is that the device is not tolerated by patients. There are many reported reasons for this noncompliance [15–21, 42]. Since the SomnuSeal mask is placed in the mouth and not on the nose, it eliminates potential limiting factors such as skin abrasions or eruptions due to pressure exerted by nasal masks, mask pressure on the ridge of the nose, eye irritation due to air leaks, dry nose, nasal stuffiness, epistaxis, and potentially sinusitis. It is less cumbersome so even claustrophobia may not be as dramatic as it is with nasal mask. Thus, it is plausible that, in the 22% of patients who complied with the oral SomnuSeal mask and not with nasal mask, these were the limiting factors. Obviously, 22% of patients are not enough, but it should be kept in mind that the participants in this study were otherwise not treated at all! Thus, 22% is a substantially positive number.

The finding that a lower pressure is required for the SomnuSeal mask compared to the nasal mask is very interesting and not intuitive. Potential explanations for this finding consist of stabilizing respiration, and maybe even more relevant, a forward displacement of the lower jaw as occurs with oral appliances [43]. In the current study we did not perform PSG studies on the treated patients and did not assess hypercapnia or respiratory control variables. As a result, we can only speculate that respiratory stabilization had occurred. In addition, we have not assessed anatomical changes of jaw position, and thus the potential anterior mandibular displacement with the SomnuSeal mask is just a speculation. Regardless of the reason for improvement in respiration with relatively low pressure, it may have beneficial effects on the cardiovascular system. Bradley et al. [44], in a study of CPAP in congestive heart failure, showed that, at a CPAP of 5 cmH_2_O, the cardiac index and stroke volume indices were increased in the subgroup with poor baseline hemodynamics and higher LV diastolic pressures [44]. Clearly, long-term studies to show any difference in cardiac function with differing CPAP pressures are needed, but it is reasonable to believe that reduction in the required optimal CPAP pressure as observed with the SomnuSeal mask may be beneficial for patients with OSA, especially those with cardiac dysfunction.

Our study has several limitations. Firstly, from the efficacy point of view, our study was a home and not a lab study. Although not tested with oral masks, but with nasal masks, there are very strong data indicating that the automatic algorithm of the CPAP is very accurate and that most patients sleeping with autoPAP have an AHI of less than 10/h. In order to reduce costs, we conducted our study in home and relied on the residual respiratory event counter of the CPAP itself. Obviously, this is a limitation, and future studies will need to be conducted in the lab. Secondly, we did not quantify outcome measures such as vigilance, mood, cognitive function, or medical outcome such as blood pressure or glucose control. This was beyond the scope of our study. The primary aim was to examine the tolerability and compliance of treated patients with this oral mask. Future studies will have to deal with behavioral medical and cognitive outcome with this interface. Thirdly, as with many previous CPAP compliance studies, this was a specific clinical study and not a field study. It is plausible that some of our patients may have made an extra effort to tolerate the SomnuSeal and it does not guarantee that they would have complied with this device in a real-life field setting. Such a study would be possible only if patients start using the SomnuSeal interface on a clinical basis. Finally, the *n* of this study is not huge. For a CPAP compliance study, more than 11 users (of 50 potential users) would be needed. We consider this study as a preliminary study with encouraging results. 

In conclusion, despite these limitations, this prospective open study of 50 noncompliant patients demonstrated that a new self-adapting mask has encouraging results in this challenging group of patients. The finding of lower than average CPAP pressures may confer important long-term cardiovascular benefits.

## Figures and Tables

**Table 1:** Data categorized according to compliance with CPAP.

	Compliant	Strugglers	Noncompliant	All
*N* (% of all)	11 (22)	5 (10)	34 (68)	50 (100)
Number of males (%)	8 (73)	5 (100)	28 (82)	41 (82)
Age	61 ± 11	49 ± 9	56 ± 13	57 ± 12
BMI	32.3 ± 4.2	37.5 ± 4.8	33.3 ± 5.0	33.6 ± 4.9
Pretreatment AHI (/h)	40 ± 19	52 ± 34	49 ± 22	47 ± 23
Pretreatment minimal O2 Sat	74 ± 11	77 ± 4	77 ± 9	76 ± 9
Days of usage	26 ± 5	13 ± 7	5 ± 5	11 ± 10
Average usage per night (h)	4.7 ± 1.1	2.9 ± 1.1	1.8 ± 0.8	2.6 ± 1.5
Residual obstructive AHI (/h)	1.9 ± 4.1	1.2 ± 2.8	n/a	n/a
Average heart rate	65 ± 12	62 ± 14	67 ± 15	66 ± 14
Total sleep time (min)	346 ± 32	359 ± 39	332 ± 30	338 ± 32
Sleep efficiency	77 ± 9	81 ± 12	75 ± 10	76 ± 9
Stage 2 (% of TST)	66 ± 19	60 ± 15	64 ± 17	64 ± 17
Stage 3 (% of TST)	18 ± 6	22 ± 7	19 ± 5	19 ± 6
REM sleep (% of TST)	16 ± 4	18 ± 5	17 ± 4	17 ± 4

**Table 2:** Results from the satisfaction questionnaire. In all questions, scale runs between 1 (weak, bad) and 5 (strong, excellent).

	Compliant	Strugglers	Noncompliant	All
Air seal with the SomnuSeal	5 ± 0	5 ± 0	4.8 ± 0.6	4.9 ± 0.5
Quality of airflow	5 ± 0	5 ± 0	4.9 ± 0.2	4.9 ± 0.1
Excess salivation	1.5 ± 0.7	2.0 ± 1.0	3.9 ± 0.9	3.1 ± 1.4
Comparison with previous tried masks (higher is better)	4.6 ± 0.5	4.0 ± 0.8	2.2 ± 0.7	3.1 ± 1.3
Willingness to purchase (higher is better)	4.0 ± 1.0	3.0 ± 0.7	1.7 ± 0.7	2.5 ± 1.3
